# Management and outcomes in patients with *Staphylococcus aureus* bacteremia after implementation of mandatory infectious diseases consult: a before/after study

**DOI:** 10.1186/s12879-015-1296-y

**Published:** 2015-12-15

**Authors:** Leslie Martin, Miriam Tova Harris, Annie Brooks, Cheryl Main, Dominik Mertz

**Affiliations:** Department of Medicine, McMaster University, Hamilton, ON Canada; Department of Medicine, University of British Columbia, Vancouver, BC Canada; Hamilton Health Sciences, Juravinski Hospital and Cancer Center, 711 Concession Street, Section M, Level 1, Room 3, Hamilton, ON L8V 1C3 Canada; Department of Pathology and Laboratory Medicine, McMaster University, Hamilton, ON Canada; Department of Clinical Epidemiology and Biostatistics, McMaster University, Hamilton, ON Canada; Michael G. DeGroote, Institute for Infectious Diseases Research, McMaster University, Hamilton, ON Canada

**Keywords:** *Staphylococcus aureus*, Bacteremia, Consultation, Outcome, Policy, Stewardship

## Abstract

**Background:**

Infectious disease (ID) consultations have been shown to increase adherence to guidelines and decrease mortality for patients with *Staphylococcus aureus* bacteremia (SAB). Here, we assessed the impact of a mandatory ID consultation policy for SAB.

**Methods:**

We retrospectively reviewed all consecutive adult patients with SAB at two tertiary care teaching hospitals in Hamilton, ON, Canada. Mandatory ID consults for SAB were implemented on January 1^st^ 2012. We compared SAB cases in 2011 (control group) with those in 2012 (intervention group). Outcomes included adherence to the Infectious Diseases Society of America guidelines and patient outcomes.

**Results:**

We reviewed 128 SAB cases in 2011 and 124 in 2012. The majority of *S. aureus* were methicillin-susceptible (97/128, 75.8 % in 2011 and 100/124, 80.6 % in 2012). ID involvement increased significantly from 93/128 (72.7 %) in 2011, to 103/124 (83.1 %) in 2012 (odds ratio [OR] 1.9, 95 % confidence interval [CI] 1.1–3.3, *p* = 0.047). There was also a significant decrease in the median time to ID involvement from 2 days to 1 (*p* = 0.001). In patients who survived the minimum treatment course (greater than 13 days), there was a significant improvement in adherence to IDSA guidelines in 2012 (65/102, 63.7 % vs. 77/96, 80.2 %; OR 2.3, 95 % CI 1.2–4.4, *p* = 0.01). Mortality and SAB relapse rates were similar in both groups.

**Conclusions:**

Creating an automated ID consultation for SAB led to an increase in involvement of ID, a significant decrease in time to ID involvement, and better adherence to IDSA guidelines. The study was not sufficiently powered to detect significant changes in mortality and SAB relapse rates.

**Electronic supplementary material:**

The online version of this article (doi:10.1186/s12879-015-1296-y) contains supplementary material, which is available to authorized users.

## Background

*Staphylococcus aureus* (*S. aureus*) bacteremia (SAB) is well described to cause significant cost, morbidity and mortality [1–6]. Current guidelines, including the guidelines by the Infectious Diseases Society of America (IDSA), detail the need for immediate intervention and further diagnostic evaluation when SAB is identified [7–9]. These include: appropriate choice and duration of antimicrobial therapy, evaluation for metastatic infection including endocarditis and the removal of infected foci. When conducted in a timely fashion these strategies have been shown to improve morbidity and mortality [2, 3, 7, 9–18]. Prior studies have illustrated that involvement of the Infectious Disease (ID) consultation service increases adherence to evidence-based treatment [2–4, 12–18] and in a number of studies has led to decreased mortality [2, 15, 16, 18 ]. Apart from a few studies, ID involvement has been at the discretion of the most responsible physician, and therefore their findings may be affected by a selection (i.e. referral) bias [14–17].

The purpose of this study was to determine whether a policy mandating an automatic ID consultation in patients with SAB improves adherence to evidence-based guidelines and patient outcomes.

## Methods

This study was conducted at two tertiary care teaching hospital sites of Hamilton Health Sciences, in Hamilton, Ontario, Canada. Patients with positive cultures from these sites were flagged at the level of the laboratory and collected for screening for enrollment in the study. Ethics approval was received from the Hamilton Integrated Research Ethics Board.

All consecutive adult patients (≥18 years of age) with an episode of SAB defined as the isolation of *S. aureus* from one or more blood culture samples were included [7, 8]. Only the first episode of SAB during the study period was included. We excluded patients in whom we could not obtain information about their therapy due to transfer to another hospital, outpatient cultures with no documentation of therapy, positive cultures from pathology, and patients who left the hospital against medical advice early in their therapy.

In this non-controlled quasi-experimental study, we included positive cultures meeting inclusion criteria from January 1, 2011 to December 31, 2011 as the control group prior to the implementation of an automated infectious disease consultation policy. The intervention group included positive cultures meeting inclusion criteria from January 1, 2012 to December 31, 2012. The policy implemented on January 1^st^ 2012 mandated ID consultation for all in-patients with SAB. The microbiology laboratory technologist reported detection of any positive blood culture for *S. aureus* to the most responsible physician and directly to the ID physician on call. The ID service was required to assess the current antibiotic treatment of in-patients with SAB within one hour and to complete a formal consult within 24 h of notification to guide management. Management was to follow an evidence-based approach as summarized in the policy, and ID was expected to follow the patient until there was complete resolution or patient discharge.

The primary outcome was pre-specified as adherence to guidelines in the management of SAB, as outlined in the internal policy, which was based on published guidelines [7, 8]. This outcome was defined as a composite of appropriate duration, dose, route, and spectrum of antibiotics, obtaining follow-up blood cultures within 3 days, completion of a TEE and/or TTE, and obtaining source control when indicated. Correct duration of antibiotics was defined as 14 +/− 2 days for uncomplicated SAB or 28-42 +/− 2 days for complicated SAB [7, 8]. Antibiotic duration was only counted as appropriate if clinicians had selected an antibiotic that would adequately treat *S. aureus* as per the guideline and policy, inclusive of cefazolin, cloxacillin, vancomycin, or daptomycin.

Secondary outcomes included in-hospital mortality, clearance of SAB defined as no relapse of SAB within 30 days after discontinuing antibiotics, re-admission defined as hospital admission within 30 days of discharge, and time to ID involvement. Patients discharged on antibiotics were deemed to have cleared SAB unless they were re-admitted with a relapse of SAB.

Categorical data was analyzed using Chi-square, non-categorical data using Student’s *T*-test or Mann–Whitney *U*-test as appropriate (PASW Version 18, Chicago, IL).

## Results

**Fig. 1:**
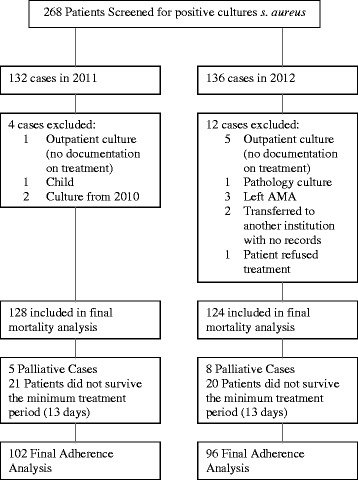
Study inclusion and outcomes. Abbreviations: *s. aureus: Staphylococcus aureus;* AMA: against medical advice

A total of 128 cases of *S. aureus* bacteremia from 2011 and 124 from 2012 were included (Fig. 1). The majority of these cases were methicillin-susceptible (MSSA; 75.8 and 80.6 % respectively, Table 1). Both centers combined, the rate of ID consultation increased significantly between 2011 and 2012 from 72.7 to 83.1 % (odds ratio [OR] 1.9, 95 % confidence interval [CI] 1.1–3.3, *p* = 0.047), an effect that was driven by the one center with a low ID involvement rate at baseline (hospital 2, 54.2 to 78.3 %, OR 3.0, 1.4–10.0, *p* = 0.005). The time to consultation was significantly reduced from 2 days to 1 day (*p* = 0.001).

**Table 1:** Patient characteristics of the baseline and intervention patient population

	2011 (Baseline)	2012 (Intervention)	Odds ratio (95 % CI)	P value
Total *n*	128	124		
Age	63.4	62.8		0.29
Male	74 (57.8 %)	74 (59.7 %)	1.1 (0.7–1.7)	0.76
Hospital 1 (vs. Hospital 2)	69 (53.9 %)	64 (51.6 %)	0.9 (0.6–1.5)	0.71
MSSA (vs. MRSA)	97 (75.8 %)	100 (80.6 %)	1.3 (0.7–2.4)	0.35
Palliative	5 (3.9 %)	8 (6.5 %)	1.7 (0.5–5.3)	0.36
ID Involvement				
Overall	93/128 (72.7 %)	103/124 (83.1 %)	1.9 (1.1–3.3)	0.047
Hospital 1	61/69 (88.4 %)	56/64 (87.5 %)	0.9 (0.3–2.5)	0.87
Hospital 2	32/59 (54.2 %)	47/60 (78.3 %)	3.0 (1.4–10.0)	0.005
Time to ID Involvement				
Days, median (IQR)	2 (1–3)	1 (1–2)	n/a	0.001
Mortality				
Overall	37/128 (28.9 %)	35/124 (28.2 %)	0.97 (0.6–1.7)	0.91
Palliative cases excluded	34/123 (27.6 %)	31/116 (26.7 %)	0.96 (0.5–1.7)	0.87
Relapse	0	0	n/a	n/a
Readmission	1/128 (0.01 %)	1/124 (0.01 %)	1.0 (0.6–16.7)	0.98

Abbreviations: *MSSA* methicillin susceptible *Staphylococcus aureus*, *MRSA* methicillin Resistant *Staphylococcus aureus*, *CI* confidence interval, *ID* Infectious Disease, *IQR* interquartile range, *n/a* not applicable

Overall, adherence to the guidelines improved from 63.7 to 80.2 % (OR 2.3, 95 % CI 1.2–4.4, *p* = 0.01) in patients that survived the minimum treatment course (greater than 13 days). Again, this result was driven by the hospital with the lower baseline adherence (57.4 to 78.3 %, OR 2.7, 95 % CI 1.1–6.6, *p* = 0.03) (Table 2). The primary cause of deviation from guidelines included: the lack of TTE or TEE (14.7 % in 2011, 7.3 % in 2012) and the lack of follow up blood cultures (12.7 % in 2011, 10.4 % in 2012). The proportion of patients with guideline-incongruent antibiotics treatment duration improved from 2011 to 2012 (11.8 % in 2011 to 3.1 % in 2012, OR 0.24, 95 % CI 0.07–0.88, *p* = 0.02). There was a trend towards lower rates of TTE/TEE in hospital-acquired cases, defined as positive blood cultures on day 3 of hospital admission or later: TTE/TEE was conducted in 121/129 of community-acquired SAB cases in both 2011 and 2012 (93.8 %) in comparison to 55/69 (79.7 %) of hospital-acquired SAB. Of note, the proportion of cases with non-adherence to more than one guideline recommendation decreased significantly from 2011 to 2012 (11.8 to 3.1 %, OR 0.24, 95 % CI 0.07–0.89, *p* = 0.02). The ID consultation service was involved in 92.3 % of cases in 2011 and 93.5 % of cases in 2012 in which there was adherence to guidelines.

**Table 2:** Outcomes of the control and intervention patient population in non-palliative patients who survived the minimum treatment period (at least 13 days)

Adherence to IDSA Guidelines				
	2011 (Baseline)	2012 (Intervention)	Odds ratio (95 % CI)	P value
Overall	65/102 (63.7 %)	77/96 (80.2 %)	2.3 (1.2–4.4)	0.01
Hospital 1	38/55 (69.1 %)	41/50 (82 %)	2.1 (0.8–5.1)	0.12
Hospital 2	27/47 (57.4 %)	36/46 (78.3 %)	2.7 (1.1–6.6)	0.03
Reason for non-adherence to guidelines				
No TTE	15/102 (14.7 %)	7/96 (7.3 %)	0.46 (0.18–1.17)	0.10
No Follow-up cultures	13/102 (12.7 %)	10/96 (10.4 %)	0.80 (0.33–1.91)	0.61
Inappropriate Antibiotic Duration	12/102 (11.8 %)	3/96 (3.1 %)	0.24 (0.07–0.88)	0.02
No source control (cases with source)	1/66 (1.5 %)	0/61 (0.0 %)	0.99 (0.96–1.01)	0.34
Greater than one of the above reasons for non–adherence to guidelines	12/102 (11.8 %)	3/96 (3.1 %)	0.24 (0.07–0.89)	0.02

Abbreviations: *IDSA* infectious disease society of America, *CI* confidence interval, *TTE* trans-thoracic echocardiogram

Despite the notable difference in adherence to guidelines when comparing the 2 years, we did not detect a difference in mortality once palliative cases were excluded (Table 1: 27.6 % in 2011 and 26.7 % in 2012, OR 0.96, 95 % CI 0.5–1.7, *p* = 0.87). A survival analysis showed no difference in the time to death comparing the two groups (Log Rank test, *p* = 0.674). There were no relapses and only two SAB-related re-admissions, thus no difference between the control and intervention group could be found.

In terms of choice of antibiotics, the proportion of patients who did not receive empiric vancomycin in addition to cefazolin or cloxacillin decreased significantly between 2011 and 2012 (13.7-3.1 %, OR 0.20, 95 % CI 0.06–0.73, *p* = 0.01 (Table 3). A total of 72 patients (28.6 %) received therapeutic courses of vancomycin for 3 days or more. Of those patients, the average course of vancomycin was 14.5 days and the average time to the first trough level was 3.14 days. The first trough level was therapeutic (15–20) in only 8 patients (11.1 %), significantly sub-therapeutic (<10) in 23 (31.9 %), and too high (>20) in 12 cases (16.7 %). Similarly, of those that remained on vancomycin long enough to receive a third trough level, 8 patients (25.8 %) were therapeutic with trough levels 15–20, whereas trough levels were too high (>20) in 18 patients (58.1 %). This highlights the challenge in achieving and maintaining therapeutic trough levels when treating patients with vancomycin.

**Table 3:** Empiric therapy used in treatment of suspected SAB

Empiric therapy	2011 (Baseline)	2012 (Intervention)	Odds ratio (95 % CI)	P value
Solely Cefazolin or Cloxacillin	14/102 (13.7 %)	3/96 (3.1 %)	0.20 (0.06–0.73)	0.01
Solely Vancomycin	32/102 (31.4 %)	36/96 (37.5 %)	1.31 (0.73–2.36)	0.36
Vancomycin + Cefazolin/Cloxacillin	14/102 (13.7 %)	22/96 (22.9 %)	1.87 (0.89–3.91)	0.09
Vancomycin + other beta-lactam	30/102 (29.4 %)	24/96 (25 %)	0.80 (0.43–1.50)	0.49
No therapy	2/102 (2.0 %)	0/96	0.98 (0.96–1.01)	0.17
Other empiric therapy	10/102 (9.8 %)	11/96 (11.5 %)	1.19 (0.48–2.45)	0.71

Abbreviation: *CI* confidence interval

## Discussion

We found an increase in adherence to published guidelines in the management of *S. aureus* bacteremia upon the initiation of an automated infectious disease consultation. However, there was no statistical improvement in patient outcomes.

There was a particularly high rate of baseline ID involvement when compared to other published studies. Jenkins et al. performed a similar before/after study in 2005 and had a baseline consultation rate of 53 % [14], whereas, in our study ID involvement was 54.2 and 88.4 % at our two hospital sites, respectively, prior to the intervention (overall rate 72.7 %). It is unclear why the rate of baseline ID involvement was significantly higher at one of our hospitals. It is possible that the practice pattern dictated this difference. As well, there are two distinct ID services at the hospital with higher baseline consultation rates (an immune-compromised and immune-competent service), which may increase accessibility to ID consultation. Despite the considerable rate of ID involvement at baseline, this study illustrated a significant reduction in time to ID consultation at the two sites combined (2 days in comparison to 1 day, *p* = 0.001). Overall, this highlights that the impact of a mandatory ID consult policy is dependent upon the practice pattern at the hospital where it is initiated.

The increase in adherence to guidelines between 2011 and 2012 was 16.4 % while the increase in ID consultation was 10.4 %. It is possible that the institution of a mandated consultation led to increased awareness around guideline-based management of *s. aureus* bacteremia both by the ID physicians as well as the most responsible physicians, which would have contributed further to the overall improvement in adherence to guidelines.

Following the intervention, it is unclear why the rate of ID involvement did not increase to one hundred percent, as would have been expected. Seven patients passed away within 72 h and five patients were palliated, which likely explains the lack of formal consultation. We cannot find an explanation for the remaining nine cases, however. It is possible that the staff physician reviewed the antibiotics and management with the care team upon being notified by the lab and never completed the formal consult, or planned to complete the formal consult however details were lost in handover.

Multiple previous studies have illustrated a mortality benefit from a similar intervention [2, 15, 16, 18]. The lack of difference in mortality in our study may be explained partially by our small sample size and potential confounding factors. Further, the overall high baseline rate of ID involvement and the relatively high level of adherence to guidelines at baseline may mask a potential effect. For example, in the study by Nagao et al. the baseline rate of TTE/TEE use prior to ID involvement was 37.1 % [15] and Jenkins et al. found a baseline rate of 57 % [14]. In our study the baseline use of echocardiogram prior to the intervention was 85.3 %. We found a very high rate of echocardiogram completion in community-acquired cases (93.8 %) with a lower rate (79.7 %) in hospital-acquired cases.

Our study differs from a number of previous studies published on this subject (Additional file 1: Table S1). Bai et al. and Fowler et al. had a similar study question, however ID consultations were offered rather than mandated thus could have led to a selection bias [4, 18]. Nagao et al. similarly developed a mandatory consultation process, however in Japan they note that ID consultation was rare for bloodstream infections and that the ID staff could not prescribe antibiotics themselves [15]. Jenkin et al. created a mandatory consultation, however, their results were underpowered and they note that there was no specific algorithm for the evaluation and management of SAB at the time of the study [14]. Most recently, Saunderson et al. published a study with mandatory ID consultations in SAB [17]. Their control arm prior to the mandatory ID consult had the benefit of phone advice from a microbiologist, which is not standard practice in many institutions.

Thus, our study offers a different perspective on a mandatory ID consultation policy. The main limitation of our study was the retrospective design with data collection from a chart review, which provides a potential for immeasurable confounding factors between the before and after patient population and relies on clear documentation from the healthcare team.

## Conclusion

The institution of an automated ID consultation for SAB led to an increase in involvement of ID, a significant decrease in time to ID involvement, and improved adherence to IDSA guidelines. However, we did not detect a difference in mortality due to this intervention in our study. Our study further adds to the growing volume of published evidence demonstrating improvement in management of SAB when ID services are involved.

## Additional file

Additional file 1:
**Overview of studies evaluating the impact of antimicrobial stewardship on management and outcomes of** ***Staphylococcus aureus*** **bacteremia.** (DOCX 21 kb)

## Abbreviations

CI: Confidence interval
ID: Infectious disease
IDSA: Infectious diseases society of America
IQR: Interquartile range
MRSA: Methicillin resistant *Staphylococcus* aureus
MSSA: Methicillin susceptible *Staphylococcus aureus*
n/a: not applicable
OR: Odds ratio
*S. aureus*: *Staphylococcus aureus*
SAB: *Staphylococcus aureus* bacteremia
