# Supplementary material for: Management and outcomes in patients with Staphylococcus aureus bacteremia after implementation of mandatory infectious diseases consult: a before/after study
Source: BMC Infect Dis. 2015 Dec 15;15:568. doi: 10.1186/s12879-015-1296-y (PMC4678701; doi:10.1186/s12879-015-1296-y)
Supplement: Additional file 1: — Overview of studies evaluating the impact of antimicrobial stewardship on management and outcomes of Staphylococcus aureus bacteremia. (DOCX 21 kb) [file 12879_2015_1296_MOESM1_ESM.docx]

| **Study** | **Setting** | **Design** | **Intervention** | **Outcomes** | |
| --- | --- | --- | --- | --- | --- |
|  |  |  |  | **Adherence to guidelines** | **Clinical outcomes** |
| Saunderson et. al.  May 2015 | United Kingdom  2006-2012  Single center | Quasi-experimental study | 2006-2009: Telephone advice from microbiologist  2009-2012 (intervention): Bedside ID consult. | Reduction in time to ID consultation, increased use of repeat cultures, echocardiography, longer antibiotic courses. More infected foci were identified however no difference in removal. | Trend towards reduced mortality, similar length of stay and defervescence. |
| Bai et. al.  March 2015 | Canada  2007-2010  6 hospital sites | Retrospective cohort study | No intervention (ID consultation optional). | Increased use of echocardiography, repeat cultures, and optimized antibiotic treatment.  No difference in removal of infectious foci. | Reduction in mortality. |
| Tissot et. al. May 2014 | Switzerland  2001-2010  Single center | Quasi-experimental study | 2007-2010: Same day ID consultation for MRSA bacteremia with microbiology notification  2001-2006: optional ID consultation. | Improvement in echocardiography, repeat cultures, removal of infected foci, sending vancomycin trough levels, antibiotic course. | Reduction in mortality. Led to an increase in length of stay. |
| Forsblom et. al. 2013 | Finland  2000-2002  2006-2007  Single center | Retrospective cohort study | No intervention (ID consultation optional). | Improvement in length of antibiotic treatment and detection of a deep focus of infection, no difference in echo with bedside consultation over phone consultation. | Reduction in ICU admission and mortality with bedside consultation over telephone consultation. |
| Robinson et. al. 2012 | Australia  1997-2007  Single Center | Retrospective cohort study | No intervention (ID consultation optional). | Improvement in antibiotic selection and duration, rate of echocardiography, repeat blood cultures. | Reduction in mortality. |
| Nagao et. al. 2010 | Kyoto  2002-2008  Single Center | Quasi-experimental study | Mandatory ID recommendations (could not order investigations or antibiotics) | Improvement in antibiotic selection and duration, rate of echocardiography, IE diagnosed more frequently, repeat blood cultures | Reduction in mortality. |
| Jenkins et. al. 2008 | 2004-2005  United States  Single Center | Quasi-experimental study | Mandatory ID consultation | Improvement in rate of echocardiography was significant. Trend towards significance in the removal of infectious foci, repeat blood cultures, antibiotic therapy, rate of consultation. | Reduction in late metastatic infection. |
| Lillie et. al. 2008 | 2005-2006  Two centers  United Kingdom | Retrospective review of prospectively collected data | Mandatory bedside ID consultation of all patients with bacteremia (not specifically *S. aureus*). No comparator. | Better choice of antibiotic regime, request of necessary investigations, no conclusive cost-effectiveness data. | Mortality rate during this period 19% (no comparator). |
| Fowler et. al. 1998 | 1994-1996  United States  Single Center | Cohort study | Offered management advice and an ID consultation (not mandatory). | No change in antibiotic duration. | No difference in mortality, increase in *s. aureus* clearance and decrease in relapse in those cases where ID advice was followed. |

Abbreviations: ID: infectious disease; *s. aureus*: staphylococcus aureus; IE: infective endocarditis
